# Screening for pathogenic neuronal autoantibodies in serum and CSF of patients with first-episode psychosis

**DOI:** 10.1038/s41398-021-01701-3

**Published:** 2021-11-05

**Authors:** Jakob Theorell, Melanie Ramberger, Ruby Harrison, Victor Mgbachi, Leslie Jacobson, Patrick Waters, Sophie Erhardt, Carl M. Sellgren, Simon Cervenka, Fredrik Piehl, Sarosh R. Irani

**Affiliations:** 1 Oxford Autoimmune Neurology Group, Nuffield Department of Clinical Neurosciences, University of Oxford, Oxford, UK; 2 Department of Clinical Neuroscience, Centre for Molecular Medicine, Karolinska Institute, Karolinska University Hospital, 171 76 Stockholm, Sweden; 3 Stockholm Health Care Services, Region Stockholm, Stockholm, Sweden; 4 Department of Physiology and Pharmacology, Karolinska Institutet, Stockholm, Sweden; 5 Centre for Psychiatry Research, Department of Clinical Neuroscience, Karolinska Institutet, Solna, Sweden; 6 Department of Neuroscience, Uppsala University, Uppsala, Sweden

**Keywords:** Diagnostic markers, Schizophrenia

## Abstract

Patients with autoimmune encephalitides, especially those with antibodies to the *N*-methyl-d-aspartate receptor (NMDAR), often present with prominent psychosis and respond well to immunotherapies. Although most patients progress to develop various neurological symptoms, it has been hypothesised that a subgroup of patients with first-episode psychosis (FEP) suffer from a *forme fruste* of autoimmune encephalitis. Without accurate identification, this immunotherapy-responsive subgroup may be denied disease-modifying treatments. Thirty studies addressing aspects of this hypothesis were identified in a systematic review. Amongst other shortcomings, 15/30 reported no control group and only 6/30 determined cerebrospinal fluid (CSF) autoantibodies. To ourselves address these—and other—limitations, we investigated a prospectively ascertained clinically well-characterised cohort of 71 FEP patients without traditional neurological features, and 48 healthy controls. Serum and CSF were tested for autoantibodies against seven neuronal surface autoantigens using live cell-based assays. These identified 3/71 (4%) patient sera with weak binding to either contactin-associated protein-like 2, the NMDAR or glycine receptor versus no binding from 48 control samples (*p* = 0.28, Fisher’s test). The three seropositive individuals showed no CSF autoantibodies and no differences from the autoantibody-negative patients in their clinical phenotypes, or across multiple parameters of peripheral and central inflammation. All individuals were negative for CSF NMDAR antibodies. In conclusion, *formes frustes* of autoimmune encephalitis are not prevalent among FEP patients admitted to psychiatric care. Our findings do not support screening for neuronal surface autoantibodies in unselected psychotic patients.

## Introduction

In recent years, a group of immunotherapy-responsive autoimmune encephalitis (AE) syndromes have been discovered, which are associated with autoantibodies that target the extracellular domains of neuronal surface proteins [1–3]. This characteristic enables autoantibody binding to neurons and glia in vivo and thus confers their likely pathogenicity. Patients with these autoantibodies typically develop amnesia, seizures and psychiatric symptoms. In some patients, particularly early in the course of *N*-methyl-d-aspartate receptor antibody encephalitis (NMDAR-Ab-E), a complex mixture of transdiagnostic psychiatric features dominate the phenotype [4, 5]. Although patients typically also present with more classically neurological features such as headache, disorientation, catatonia, hyperkinesis and speech deficits [2, 5–7], the existence of *formes frustes* of AE has been proposed, especially in patients who display exclusive psychiatric symptoms [8, 9]. Importantly, such patients could be misdiagnosed with primary psychiatric illnesses and hence denied effective immunotherapies.

In this study, we address the hypothesis that some patients with pure psychiatric features have *formes frustes* of AE. First, we conducted a systematic review of the literature to identify methodological inconsistencies and limitations from previous attempts to address this question. Next, we designed a study to address many of the identified limitations: live cell-based assay (CBA) neuronal surface protein autoantibody screening was performed in serum and paired cerebrospinal fluids (CSFs) from a well-characterised incident cohort of first-episode psychosis patients and age and sex-matched healthy controls.

## Methods

### Systematic review

Searches for full-text MEDLINE English articles with human subjects from 2007 to July 2020 were made on PubMed with the following search terms (number of hits in brackets):

- “autoantibodies/autoantibody psychosis” (319)
- “NMDAR psychosis” (334)
- “NMDA autoantibody psychosis” (93)
- “neuronal autoantibody schizophrenia” (36)

The following search terms were used for Google scholar, where only a restriction to studies from 2007 and onward was set (the first 300 most relevant hits investigated in each case):

- “NMDA autoantibody psychosis” (3500)
- “neuronal autoantibody psychosis” (5770)
- “neuronal autoantibody schizophrenia” (5720)

Case reports were excluded, as were studies using other modes of antibody detection, such as enzyme-linked immunoassay. For statistical analyses, Hammer et al. [10] were excluded, as this dataset was included within the larger study of Dahm et al. study [11]. Only studies including both patients and controls were included (in bold, Table 1).

**Table 1:** Summary of published data reporting autoantibodies in primary psychiatric cohorts.

Group	First author	Year	FEP^a^	NMDAR-IgG in serum^b^				CBA^c^	Lab^d^	Other Abs^e^	NMDAR-IgG in CSF	Neurological symptoms
				Patients		Controls						
				–	+ (%)	–	+ (%)					
–	This study	2020	Yes	71	1 (1.4)	47	0 (0)	Live	Yes	6	0/117	0
1	**Pathmanadavel**	2015	Yes	35	8 (22.9)	43	0 (0)	Live	No	1	NA	0
1	**Lennox**	2017	Yes	208	20 (9.6)	101	4 (4.0)	Live	No	5	NA	0
1	**Jezequel**	2017	No	39	9 (23.1)	101	3 (3.0)	Live	No	0	NA	0
2, 4	**Steiner**	2013	No	119	2 (1.7)	230	0 (0)	Fixed	Yes	1	2/4	2/2
2	Bergink	2015	No	92	4 (4.3)	64	0 (0)	Fixed	No	8	NA	0
3	**Masdeu**	2012	Yes	80	0 (0)	40	0 (0)	Fixed	No	0	NA	NA
3	**Masopust**	2016	Yes	50	0 (0)	50	0 (0)	Fixed	No	0	NA	NA
3	**Mantere**	2016	Yes	70	0 (0)	34	0 (0)	Fixed	No	12	NA	NA
3	**Gaughran**	2018	Yes	95	1 (1.1)	97	1 (1.0)	Live	No	3	NA	0
3	**Hermán**	2020	Yes	37	0 (0)	21	0 (0)	Fixed	No	0	NA	NA
3	**Rhoads**	2011	No	7	0 (0)	3	0 (0)	Fixed	No	0	NA	NA
3	Hammer	2014	No	1074	7 (0.7)	1267	5 (0.4)	Fixed	No	0	NA	NA
3	**Blackburn**	2020	No	66	2 (3.0)	35	1 (2.9)	Fixed	No	5	NA	NA
3	**Hoffmann**	2020	No	67	0 (0)	27	0 (0)	Fixed	No	5	NA	0
3	**Dahm**	2014	No	1370	8 (0.6)	1683	20 (1.2)	Fixed	No	11	NA	NA
4	Endres	2015	Yes	NA	NA	NA	NA	Both	Yes	3	1/125	1/1
4	Scott	2018	Yes	109	4 (3.7)	NA	NA	Fixed	No	3	3 /113	2/3
4	Tang	2019	Yes	11	4 (36.4)	NA	NA	Live	Yes	1	3/3	3/3
4	Kelleher	2015	Yes	81	4 (4.9)	NA	NA	Live	No	0	1/85	1/1
4	Tsutsui	2012	No	51	10 (19.6)	NA	NA	Fixed	No	0	NA	9/10
–	Ando	2016	Yes/no	17/36	4/2 (23.5/5.6)	NA	NA	Fixed	No	0	NA	0
–	Chen	2017	Yes/no	78/234	0 (0)	NA	NA	Fixed	No	4	NA	NA
–	Zandi	2011	Yes	44	2 (4.5)	NA	NA	Live	No	1	NA	0
–	Oviedo-Salcedo	2018	Yes	121	3 (2.5)	NA	NA	Fixed	Yes	3	0/124	NA
–	Haussleiter	2012	No	50	0 (0)	NA	NA	Fixed	No	6	NA	NA
–	Van Mierlo	2015	No	104	0 (0)	NA	NA	Fixed	No	24	NA	NA
–	Schou	2016	No	144	0 (0)	NA	NA	Fixed	No	4	NA	NA
–	De Witte	2015	No	475	0 (0)	NA	NA	Fixed	No	0	NA	NA
–	Beck	2015	No	40	3 (7.5)	NA	NA	Live	No	0	NA	0
–	Jezequel	2017	Yes	289	9 (3.1)	NA	NA	Live	No	0	NA	NA

Studies are grouped by those which reported higher IgG seropositivity rates for autoantibodies in psychiatric patients with statistical significance (Group 1) or statistically non-significant trends (Group 2) or no differences (Group 3). Group 4 indicates those without control groups who do describe neurological features in the cohort. Bold names indicate inclusion in Fisher’s exact test for comparison of the sensitivity of the live and fixed cell-based assay (CBA).

*FEP* first-episode psychosis, *Lab* laboratory analyses of immunological parameters, *CASPR2* contactin-associated protein-like 2, *LGI1* leucine-rich glioma inactivated 1, *AMPA* α-amino-3-hydroxy-5-methyl-4-isoxazolepropionic acid, *DPPX* dipeptidyl-peptidase-like protein 6, *MOG* myelin oligodendrocyte glycoprotein, *AQP4* aquaporin 4, *DNER* delta/notch-like epidermal growth factor-related receptor.

^a^The diagnoses in non-first episode psychosis studies were: schizophrenia (10), psychosis disorders (3) and acute psychosis, post-partum psychosis or refractory psychosis (1 each).

^b^In these assays, a fixed assay platform incorporates primate cerebellum and rodent hippocampal neurons, but it is generally not specified whether one or both are considered to be positive for the overall result to be positive.

^c^Differences between live and fixed CBAs are described in the Supplementary section.

^d^Other laboratory tests variably included CSF white count, IgG index/levels, albumin quotient and oligoclonal bands.

^e^Neuronal surface antibodies against antigens than the NMDAR most commonly included those directed against CASPR2, LGI1, the γ-aminobutyric acid A/B receptors, glycine receptor, AMPA receptor, DPPX, metabotropic glutamate receptor 1 and 5, MOG, AQP4, DNER, IgLON5 and dopamine receptor D2.

### Patient recruitment and diagnostic testing

First-episode psychosis patients (*n* = 74), defined as first physician contact due to psychotic symptoms, were recruited from psychiatric wards and outpatient departments (1:1 ratio) as part of the Karolinska Schizophrenia Project (ethical approval: 2010/879-31-1). Post hoc, three patients were excluded (one lacking a psychosis diagnosis, one due to suspected drug-induced psychosis and one due to prolonged antipsychotic treatment). None of the 71 remaining patients showed overt abnormal signs on neurological examination, seizures, autonomic instability or a movement disorder. Upon recruitment, all patients underwent an assessment using the Structured Clinical Interview for DSM-IV Axis I Disorders plus CSF and blood sampling. Patients fulfilled criteria for either schizophrenia (44/71; 62%), unspecified psychosis/psychosis without further specification (18/71; 25%), delusional syndrome (5/71; 7%), brief psychotic episode (3/71; 4%), schizoaffective syndrome (1/71) or depression with psychotic symptoms (1/71). At 18-month follow-up, patients fulfilled criteria for schizophrenia (*n* = 21/37; 57%) unspecified psychosis/psychosis without further specification (*n* = 5/37; 14%), schizoaffective syndrome (*n* = 2/37; 5%), a delusional syndrome (*n* = 2/37; 5%) or brief psychosis (*n* = 1/37), and 6/37 (16%) did not fulfil criteria for any psychiatric or neurologic diagnosis. Healthy controls (*n* = 48) were recruited by advertisement and underwent a physical examination, blood and urine laboratory screening and the Mini International Neuropsychiatric Interview, to exclude somatic or psychiatric pathologies. All participants were prospectively recruited between 2011 and 2017 and provided written informed consent before enrolment.

### Clinical laboratory testing

Serum and CSF were collected as previously described [12]. In brief, blood and CSF samples were collected in the morning, with separation and freezing of serum and cell-free CSF at −80 °C within 1 h. Routine blood and CSF analyses (in the Departments of Clinical Chemistry and Clinical Immunology, Karolinska Institute) included cell counts, protein concentrations, immune electrophoresis (for the presence of oligoclonal bands) and neurofilament-light concentrations (Uman Diagnostics, Umeå, Sweden).

### Autoantibody detection

For autoantibody detection, live CBAs were performed as previously described [7, 13–15]. Patient sera and CSF were incubated for 1 h with live HEK293T cells, each transiently transfected to surface express the following full-length human autoantigens: contactin-associated protein-like (CASPR2), membrane-tethered leucine-rich glioma-inactivated 1 (LGI1), the glycine receptor, dopamine 2 receptor, γ-aminobutyric acid A and B receptors and the NMDAR. To ensure highly sensitive NMDAR-antibody testing, the NR1 and NR2B subunits of the NMDAR were co-expressed using both an unmodified and a C-terminal EGFP-tagged version of the NR1 subunit (with and without the extracellular exon 5, respectively) [16]. NMDAR-reactive samples were tested against the mutated version of the NR1 subunit (N368Q), reported as an immunodominant epitope [17]. Secondary antibodies against the Fc region of human immunoglobulin G (IgG) were applied post-fixation and subsequent visualisation was performed blinded to patient/control status. Due to different levels of background binding, the starting serum dilutions have been established as 1:100 for CASPR2 and glycine receptor antibody detection, and 1:20 for other antigens. CSF testing was performed undiluted. Any positive findings were titrated to endpoint dilution. The patient/control status was only revealed after all screening and confirmatory assays had been completed. The three individuals excluded post hoc were antibody negative. The experimental design was defined before the initiation of testing: www.github.com/jtheorell/KaSP_AE.

### Statistical analyses

Fisher’s exact tests were used to compare ratios of antibody-positive subjects between cases and controls. For calculation of binomial proportion confidence intervals for AE among FEP patients, Wilson’s score interval was calculated using R and the fastR2 package [18, 19]. Uniform Manifold Approximation and Projection (UMAP) of the Positive and Negative Syndrome Scale (PANSS) item data was conducted using the UMAP function in the R uwot package [20], using standard settings (15 nearest neighbours, spectral initiation, Euclidean distances as metric). The data were not pre-transformed, scaled or centred before the generation of the UMAP, as all PANSS items are recorded on the same scale, and all differences in magnitude between them thus reflect clinical differences. Due to the low number of seropositive patients, no attempts were made to statistically compare these to the seronegative patients.

## Results

### Systematic review

To understand how comprehensively published studies had addressed the question of *formes frustes* of AE, a systematic review was conducted. The specified search terms identified 29 studies detecting neuronal surface autoantibodies by CBAs in patients with primary psychiatric diseases (3 unique to PubMed, 15 unique to Google Scholar, 11 both) [8, 10, 11, 21–46]. One additional article [47] was referenced within other studies (Table 1). From a total of 30 studies, 15 (50%) did not report traditionally neurological features and 6 of the remaining 15 included patients with clinical signs of encephalitis. No control group was studied in 15/30 (50%), only 5/30 (17%) included descriptions of concomitant inflammatory parameters and 10/30 (33%) tested for a single antigen. Ten out of 30 (33%) used live CBAs: this testing approach preferentially exposes patient IgG to native extracellular epitopes. In addition, the systematic review revealed that live CBAs were positive in 38/415 (9%) patients versus 8/350 (2%) controls (*p* < 0.0001, Fisher’s exact test), whereas the equivalent rates using fixed CBAs were 12/1866 (0.6%) and 21/2123 (0.1%), respectively (*p* = 0.29, Fisher’s exact test), suggesting the latter approach fails to differentiate patients from controls. Finally, no studies have systematically evaluated CSF samples in both patients and controls and only 6/30 (20%) studies tested NMDAR antibodies in CSF, a key sample in the accurate diagnosis of NMDAR-Ab-E. All previous studies show at least two of these limitations. To address these, an analysis of the Karolinska Schizophrenia Project cohort was undertaken.

### Cohort characteristics and autoantibody results

**Fig. 1:**
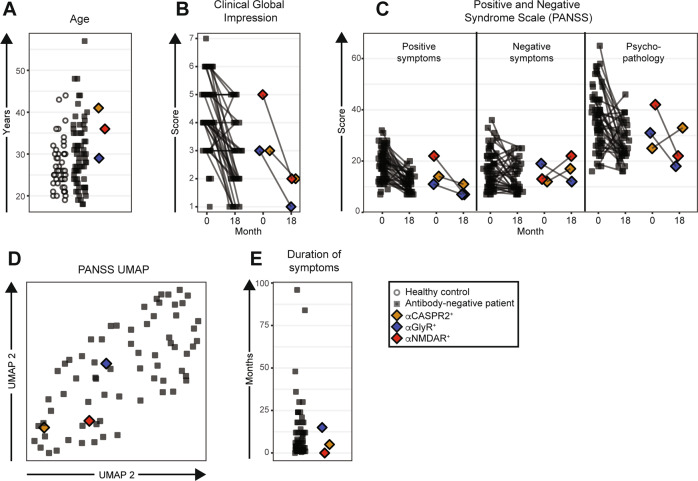
Clinical comparisons of the three seropositive first-episode psychosis patients versus control groups. Sera from three first-episode psychosis patients out of 71 displayed specific binding to either the glycine receptor (blue), NMDA receptor (red) or CASPR2 (yellow) at borderline or moderate endpoint dilutions, while none of the 48 healthy controls (white circles) displayed binding. Grey squares denote the first-episode psychosis patients without autoantibodies. **A** Age distribution. **B** Clinical Global Impression. A value of 1 corresponds to a healthy state and 7 to the most extreme psychopathology. **C** Sums of the three classes of symptoms in the Positive and Negative Syndrome Scale (PANSS). Ranges 7–49 for positive and negative symptoms and 16–112 for general psychopathology. High values indicate more symptoms. Dots connected by lines correspond to the same individual at onset and at the 18-month follow-up. **D** Uniform Manifold Approximation and Projection (UMAP) of the 30 PANSS items. This unsupervised visualisation shows the total PANSS variance. **E** Duration of self-reported symptoms.

Within this cohort, first-episode psychosis subjects and healthy controls were well matched for sex (47% of patients and 50% of healthy controls were female) and age (Fig. 1A). Patients were drug-naive (*n* = 35) or had brief exposure to antipsychotics (*n* = 37, median 13, range 2–38 days) and none showed aberrant neurological features. Electroencephalography was not performed. No healthy control sera IgG bound to any autoantigen, by comparison to 3/71 (4%) first-episode psychosis sera IgG (*p* = 0.28, Fisher’s exact test). These three showed exclusive binding to either the NMDAR, glycine receptor or CASPR2, at endpoint dilutions, which are borderline (1:40 or 1:100) or moderate (1:2000), respectively. The NMDAR-reactive IgG bound both isoforms of NR1, and its binding was abrogated when the NR1 mutant was expressed. From the CSF of these three patients, the corresponding autoantibody was not detected. In addition, as CSF NMDAR antibodies have been reported without accompanying serum NMDAR antibodies, all 119 CSF samples were tested for NMDAR antibodies—and found to be negative to both NR1 isoforms.

### Case vignettes for seropositive, CSF-negative patients

The three seropositive patients were female and showed no neurological symptoms throughout their course.

#### NMDAR autoantibody seropositive patient

This 36-year-old outpatient experienced psychotic symptoms for <4 weeks at the time of sampling, with a burden of symptoms within the range of seronegative patients (Fig. 1). Hallucinations and disorganised thinking were dominant with delusions, feelings of guilt and prominent depression. She showed no aggression or anxiety and had no seizures or movement disorder. She received benzodiazepines for a few days prior to sampling. Magnetic resonance imaging (MRI) of the brain was unremarkable. By the 18-month study follow-up, she had been diagnosed with unspecified schizophrenia and, at this timepoint, her clinical profile was dominated by blunted affect and a lack of spontaneity.

#### Glycine receptor autoantibody positive patient

This 28-year-old had suffered from psychotic symptoms for 15 months prior to the delivery of psychiatric care and hospitalisation. Olanzapine had been administered for 12 days prior to blood sampling. At this timepoint, the entry to this study, her clinical picture was of predominant negative symptoms including withdrawal and lack of spontaneity, plus persecutory delusions and conceptual disorganisation. Brain MRI was unremarkable. By 18-month follow-up, she had been diagnosed with paranoid schizophrenia. Anxiety and difficulty in abstract thinking were the most prominent features at this point (Fig. 1).

#### CASPR2 autoantibody positive patient

At the time of sampling, this 40-year-old had persecutory delusions and anxiety for 5 months and had been treated with olanzapine and benzodiazepines for 10 days as an outpatient. Brain MRI was normal. At 18 months follow-up, she had been diagnosed with unspecified schizophrenia and had developed more negative symptoms with blunted affect, a lack of spontaneity, anxiety and motor retardation.

### Comparison of seropositive and seronegative cases

**Fig. 2:**
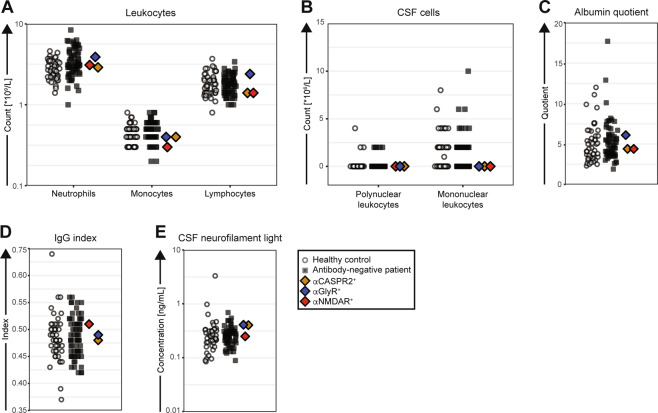
Immunological comparisons of the three seropositive first-episode psychosis patients versus control groups. **A** Blood leucocyte differential counts. **B** Polynuclear and mononuclear leucocyte counts in CSF. **C** Albumin quotient, a marker of blood–brain barrier permeability, calculated as CSF albumin/serum albumin × 100. **D** IgG index, a measure of intrathecal IgG synthesis, is defined as ([CSF IgG/serum IgG] × [serum albumin/CSF albumin]). **E** Neurofilament light-chain levels in CSF, a marker of axonal damage.

Overall, by comparison to seronegative cases, the three seropositive patients showed a similar age at onset (Fig. 1A) and symptom severity, as characterised with the Clinical Global Impression Scale (Fig. 1B). The seropositive patients’ positive and negative symptoms, measured by the PANSS, and their general psychopathology category sums, were within the range of percentiles 10–68, 35–62 and 15–68 of the seronegative patients, respectively. At follow-up, the same PANSS symptom category sums were in the range of percentiles 15–38, 56–85 and 12–76 of the seronegative patients, respectively (Fig. 1C). Overall, neither individual symptoms nor the overall pattern of symptoms were remarkable within the seropositive patients (Fig. 1D and Supplementary Fig.). The duration of psychotic symptoms for the patient with low serum NMDAR antibodies was among the shortest in the cohort, but the other two seropositive patients had symptom durations common among seronegative patients (Fig. 1E). Administered treatments were similar (not shown) and no patients received electroconvulsive therapy during the course of the study. Finally, when comparing markers of peripheral (Fig. 2A) and central (Fig. 2B) inflammation, blood–brain barrier leakage (Fig. 2C), intrathecal antibody production (Fig. 2D) and axonal damage (Fig. 2E), the results were similar between the seropositive and seronegative first-episode psychosis patients and healthy controls. None of the three patients fulfilled the proposed criteria for patients with a probable autoimmune cause of psychosis [9, 48, 49].

## Discussion

In this study, potentially pathogenic serum IgG autoantibodies against neuronal surface proteins were found in serum, but not CSF, from 4% of first-episode psychosis patients who lacked overt neurological features. These seropositive patients showed no distinctive clinical features and no laboratory or imaging evidence of a skewed peripheral or CSF immune response. This observation stands in striking contrast to patients with autoantibody-mediated encephalitis, who typically have highly characteristic clinical features often with inflammatory paraclinical findings. Hence, phenotypes and parameters fundamental to encephalitis and neuroinflammation were not enriched in seropositive first-episode psychosis patients, suggesting that these autoantibodies do not indicate the presence of mild encephalitis, and may represent clinically irrelevant results.

Despite detailed analyses of both the overall cohort features and qualitative individualised patient data, we observed no features of bona fide encephalitis in the three seropositive patients from this study. While this represents only a small number of seropositive individuals, our data concur with previous studies of neuronal surface autoantibody seropositive patients with primary psychiatric diagnoses, which have generally shown no enrichment for specific psychiatric or neurological features. The few exceptions identified neurological symptoms typical of AE in their seropositive cohorts (Group 4 and Table 1), likely as they often recruited acute unselected psychotic patients, a minority of whom have clear-cut features of encephalitis. Indeed, since our study closed to recruitment other groups have also suggested that NMDAR antibody screening should only be applied to the atypical ‘flagged’ psychiatric features, which, almost perfectly, overlap with those observed in patients with AE [48–55]. Two of these reports, with a total of 208 FEP patients and 40 healthy controls, also failed to identify any cases of AE [54, 55]. When the data from these two studies are combined with the current study, the Wilson test shows a maximal AE rate of 1.7% (with a 95% confidence interval) among unselected first-episode psychosis patients.

Selective screening of populations with a high pre-test probability will reduce the potential of iatrogenic harm by acting on ‘false-positive’ results. However, while it remains possible that these antibodies are not present (correctly termed ‘false positives’), it may be that they are present at low titres, or with low binding strengths. The latter possibility may explain their lack of binding at higher dilutions and to brain tissue, settings in which a single autoantigen is not actively overexpressed [45, 54, 55]. Our data support the antigen-specific nature of these low positive antibodies as individual serum IgGs only bound to one of the tested antigens and by the NMDAR antibody reactive sample whose binding was abrogated after a single point mutation to the established immunodominant NR1 subunit of the NMDAR [16]. Hence, it remains possible that these antibodies influence disease trajectories, as suggested in studies of stroke and dementia [56, 57].

An important variable across studies has been the biological sample: serum has been tested in all previously reviewed studies (Table 1). Some of these studies have reported statistically higher IgG seropositivity rates in psychiatric patients (Group 1, Table 1) and others showed similar trends without statistical significance (Group 2, Table 1). In addition, many report no differences between the groups (Group 3, Table 1), including a seropositivity rate of ~2% across disparate neuropsychiatric and neurodegenerative disease groups without encephalitis [11, 44, 56]. Our literature review identified that this variability can be partly attributed to differences in methodology, with all the studies in Group 1 being performed with live CBAs, versus fixed CBAs in all but one of the studies in Group 3 [29]. The higher sensitivity of live CBAs has been previously demonstrated for serum NMDAR autoantibody detection [22], including in a recent meta-analysis [58]. However, as serum NMDAR antibodies have been reported in ~3–10% of healthy individuals [11, 53], the diagnosis of NMDAR-Ab-E is most specifically confirmed by the identification of NMDAR autoantibodies in CSF. Whereas other autoantibodies are both most sensitively and specifically detected in serum, such as those directed against LGI1 [52]. Hence, our paired serum–CSF testing aimed to comprehensively capture known IgG autoantibodies from appropriate compartments.

None of our patients exhibited the ‘flagged’ features noted by others, confirming the very limited ‘real-world’ overlap between AE and primary psychosis. Yet, it remains possible that the selection of cohorts with higher pre-test probabilities will yield immunotherapy-responsive subsets. For example, in our study, there were no patients with clear signs of catatonia at the time of sampling. Catatonia patients often display combinations of hypokinesia, hyperkinesia and volitional abnormalities [59], and this phenomenon appears over-represented in patients with NMDAR autoantibody encephalitis [4].

In summary, our findings do not support non-selective autoantibody screening in first-episode psychosis, as *formes frustes* of AE appear rare in typical psychiatric presentations and the rate of clinically irrelevant serum positivity remains appreciable. Rather, this study supports an a priori phenotype-driven approach to testing [9, 48, 49] and simultaneous testing in serum and CSF [50], generating a higher pre-test probability and specificity and a consequent straightforward interpretation of a positive test result.

## Supplementary information

Supplementary figure, showing individual items of the Positive And Negative Syndrome Scale. For all items, a value of 1 corresponds to a healthy state and 7 to the most extreme psychopathology. Dots connected by lines correspond to the same individual at onset and at the 18-month follow-up.
