# Supplementary material for: Screening for pathogenic neuronal autoantibodies in serum and CSF of patients with first-episode psychosis
Source: Transl Psychiatry. 2021 Nov 5;11:566. doi: 10.1038/s41398-021-01701-3 (PMC8571405; doi:10.1038/s41398-021-01701-3)
Supplement: Supplementary file 1 — Supplementary figure, showing individual items of the Positive And Negative Syndrome Scale. For all items, a value of 1 corresponds to a healthy state and 7 to the most extreme psychopathology. Dots connected by lines correspond to the same individual at onset and at the 18-month follow-up. [file 41398_2021_1701_MOESM1_ESM.docx]

**Supplemental Figure**

**Individual items of the Positive And Negative Syndrome Scale.** For all items, a value of 1 corresponds to a healthy state and 7 to the most extreme psychopathology. Dots connected by lines correspond to the same individual at onset and at the 18-month follow-up.
